# A Comparison of Alternative Distributed Dynamic Cluster Formation Techniques for Industrial Wireless Sensor Networks

**DOI:** 10.3390/s16010065

**Published:** 2016-01-06

**Authors:** Mohammad Gholami, Robert W. Brennan

**Affiliations:** Department of Mechanical and Manufacturing Engineering, Schulich School of Engineering, University of Calgary, Calgary, AB T2N 1N4, Canada; gholamim@ucalgary.ca

**Keywords:** wireless sensor networks, clustering, distributed adaptive systems, reconfigurability

## Abstract

In this paper, we investigate alternative distributed clustering techniques for wireless sensor node tracking in an industrial environment. The research builds on extant work on wireless sensor node clustering by reporting on: (1) the development of a novel distributed management approach for tracking mobile nodes in an industrial wireless sensor network; and (2) an objective comparison of alternative cluster management approaches for wireless sensor networks. To perform this comparison, we focus on two main clustering approaches proposed in the literature: pre-defined clusters and *ad hoc* clusters. These approaches are compared in the context of their reconfigurability: more specifically, we investigate the trade-off between the cost and the effectiveness of competing strategies aimed at adapting to changes in the sensing environment. To support this work, we introduce three new metrics: a cost/efficiency measure, a performance measure, and a resource consumption measure. The results of our experiments show that *ad hoc* clusters adapt more readily to changes in the sensing environment, but this higher level of adaptability is at the cost of overall efficiency.

## 1. Introduction

**Figure 1:**
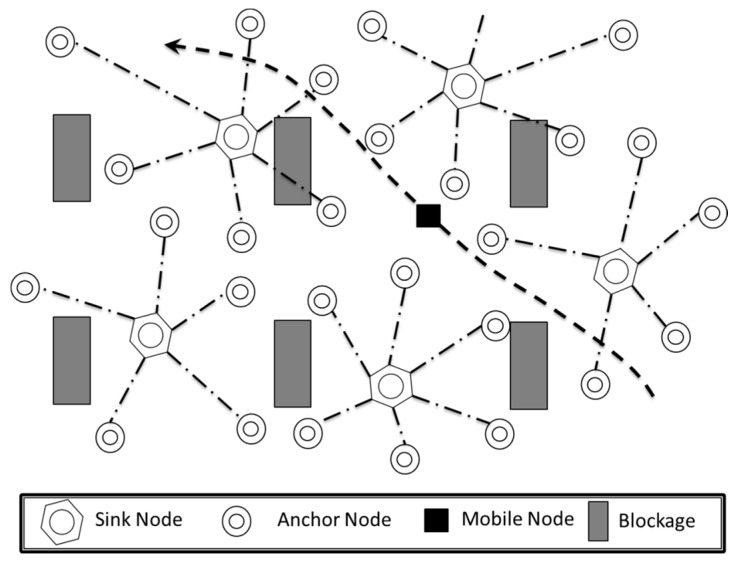
Mobile Node tracking in a factory wireless sensor network (WSN).

Since its introduction in the 1980s and 1990s for military defense applications, wireless sensor network (WSN) technology has expanded in scope to include a wide range of civilian applications such as infrastructure security, environmental and habitat monitoring, industrial sensing, and traffic control [1]. More recently, there has been considerable interest in deploying WSN technology at the lowest level of factory automation systems [2]. For example, Figure 1 shows the basic problem tackled in this paper: distributed dynamic cluster formation to track mobile wireless sensor nodes in a factory environment. In this figure, we show a set of static wireless sensor nodes (Anchor Nodes and Sink Nodes) that are used to track mobile wireless sensor nodes: the Anchor Nodes provide distance estimates of the Mobile Nodes to the Sink Nodes, where the localization calculation takes place.

Industrial wireless sensor networks (WSNs) have become a research trend due to the advances in processing power for micro-computers and reduced battery consumption of embedded battery powered devices. General WSNs are composed of wireless sensor nodes, which are small with limited processing and computing resources and are inexpensive compared to traditional sensors. Sensor nodes are used to sense, measure and gather information from the environment and transmit the data to a user or data acquisition system.

Hardware and software technologies are currently available to provide sophisticated monitoring, control, and diagnostics of industrial systems at lower costs than ever before. There are, however, a number of implementation challenges when these new technologies are applied in industrial environments. Given the distributed nature of many of these systems, management and coordination are key challenges. Reliability and robustness given harsh industrial conditions, which can arguably require higher reliability requirements than other commercial systems, is another key implementation issue.

Given the harsh, uncertain, and dynamic conditions present on the factory floor, WSNs applied in this domain must be capable of rapidly adapting to change in order to support the flexibility and responsiveness required of the system as a whole. More specifically, the WSN should be capable of allowing sensor nodes to be added and/or removed from the system on-the-fly to support changes in the configuration of the shop floor, and also be capable of handling signal loss that occurs during the tracking process as a result of blockage and/or noise from machinery.

As a result, the wireless sensor network must be dynamically reconfigurable: *i.e.*, it should have “… the ability to repeatedly change and rearrange (its components) in a cost effective way” [3]. Given the limited battery life of wireless sensor nodes, “cost effective” in this context relates to reducing the amount of communication and the corresponding energy consumption of the system.

This paper addresses this set of challenges with the following contributions. First, we develop a novel distributed management approach for tracking mobile nodes in a 400–676 node network subject to harsh industrial conditions presented by signal blockage and noise. Although Mobile Node tracking can be accomplished using a centralized WSN management approach, we propose a distributed, agent-based approach to achieve the flexibility and responsiveness required of factory automation systems. As illustrated in Figure 1, we propose dividing the factory environment into sensor zones, consisting of groups of static Anchor Nodes that are clustered around fixed Sink Nodes. Each cluster zone is managed by the Sink Node and is responsible for tracking Mobile Nodes that pass through its geographic area.

Secondly, we provide an objective comparison of alternative distributed cluster formation techniques for wireless sensor node tracking. Our focus here is to quantify the trade-offs associated with competing reconfigurable systems approaches: although various reconfigurable system designs have been proposed in the literature [3], the trade-offs associated with these alternative designs is still an open issue. To accomplish this, we focus on two main approaches proposed in the literature: (1) pre-defined clusters based on efficient geographical partitioning of sensor nodes around cluster heads; and (2) *ad hoc* clusters that are formed “on the fly” during tracking. We compare these approaches in the context of their reconfigurability, and in particular, how cost effectively they modify or adapt to meet new or changed requirements; to perform this comparison, we develop two new metrics: (1) an efficiency/cost based metric; and (2) a performance-based metric. As well, we compare the tracking performance of these approaches using a tracking accuracy metric.

In the next section, we provide a summary of related work on wireless sensor node localization and tracking. Next, we describe the WSN architecture and the clustering approaches explored in this research. In Section 4, we present our experiments with these clustering approaches then provide our conclusions and future work in Section 5.

## 2. Related Work

Individual sensors are incapable of solving the tracking task themselves, but instead must be organized in a collaborative manner that allows their individual measurements to be used to triangulate the target node. In this section, we provide an overview of the current work on how objects are located using wireless sensor nodes, and on how wireless sensor networks can be coordinated to track target nodes.

### 2.1. Wireless Sensor Network Localization

The term “localization” refers to the process of calculating a node’s physical position, or location, in space. In recent years, a variety of approaches have been proposed that fall into two general categories: range-based and range-free localization [4]. The former category refers to protocols that use absolute point-to-point distance or angle estimates to calculate the location of a wireless sensor node; the latter category refers to protocols that make no assumptions about the availability of point-to-point distance or angle estimates, but instead rely on the structure of the network.

Although range-free techniques require less sophisticated hardware than range-based techniques [5], they typically assume an isotropic network where the hop count between two nodes is proportional to their distance [6]. Given the presence of non-isotropic conditions on the shop floor resulting from signal loss due to noise and blockage and the need to reconfigure the network configuration to adapt to change, we have chosen to use range-based localization in this research.

Two commonly used approaches to estimate point-to-point distance between nodes for range-based localization are Received-Signal-Strength (RSS) and Time-of Arrival (ToA). RSS measures the power of the signal at the receiver and calculates the distance according to the propagation loss model. ToA measures the propagation time of the received signal (typically radio signals for large distances or ultrasound for small distances) and determines the distance by multiplying it by its own speed. In general, RSS is an easier parameter to implement, while ToA may achieve a higher accuracy [7]. For this research, we have chosen to use ultrasound-based ToA because of its higher accuracy, and also to align our current simulation work with our previous experimental work [8] using the Cricket platform [9].

### 2.2. Wireless Sensor Node Clustering Strategies

Clustering in wireless sensor networks is a matter of interest not only as a method of data aggregation but also as a backdrop to support other wireless sensor network protocols such as routing and localization. The approach is used to organize fixed sensor nodes, distributed over a wide geographic area, into smaller groups, or clusters, that are responsible for specific regions in the tracking zone.

The first problem that researchers must tackle when considering wireless sensor node clustering, is how these clusters are defined. Horling *et al.* [10] show the importance of this decision by comparing of the characteristics and trade-offs of different organizational types. They propose a multi-agent system design to explore geographic coalitions (*i.e.*, size and shape of the sector) and functional differentiation, and perform experiments to determine the effect of sector size on communication load, load disparity between agents, average communication distance, and quality of tracking. Their results support the use of agents for these systems, but show that further work is required to fully understanding the effects of organizational trade-offs.

Recent research on WSN clustering strategies has focused primarily on improving the efficiency of the overall network. For example, the desire to reduce the time and cost associated with replacing batteries in sensors has motivated research on partitioning the WSN in ways that minimize overall energy consumption. Bandyopadhyay and Coyle [11] propose a distributed randomized clustering algorithm for homogeneous wireless sensor networks: *i.e.*, networks where every wireless node can become a cluster head with a probability that minimizes the total energy spent in the network. Yu *et al.* [12] present an energy-efficient dynamic clustering technique to route sensed information from the field sensors to a remote base station in large-scale sensor networks. In a network of homogeneous wireless sensor nodes, a probabilistic approach is employed to determine the cluster heads.

Sun *et al.* [13] propose a distributed cluster formation technique to increase security in wireless sensor networks. The proposed distributed protocol first divides the nodes into mutually disjoint cliques. Then, all the normal nodes in each clique agree on the same clique memberships. Finally, while external attackers can be prevented from participating in the cluster formation process, inside attackers that do not follow the protocol semantics can be identified and removed from the network.

Research has also focused on efficient geographical partitioning of sensor nodes around cluster heads. One particular method of interest that has been used to define clusters is the Voronoi diagram [14]. This method divides a set of points into regions, or cells, that each contains all points that are closest to a specified “seed” location. In the context of Figure 1, seed locations correspond to sink nodes and points correspond to anchor nodes.

Chen *et al.* [15] propose a dynamic clustering technique based on Voronoi diagrams to track acoustic targets in wireless sensor networks. Given a heterogeneous wireless sensor network where wireless nodes possess different capabilities, the authors envision a hierarchical cluster that assumes the role of cluster head for the nodes with higher capabilities that are sparsely distributed in the network field. Voronoi diagrams are used by the cluster heads to muster an appropriate number of sensor nodes and dynamically form the clusters, and also to ensure only one cluster remains active at each time to track the mobile target.

Boukerche *et al.* [16] adopt a range-based approach to localize the wireless sensor networks. As such, using average size of a hop, hops are mapped to distance units. To overcome the scalability limits of a DV-hop technique that is imposed by the large communication cost, the authors propose an approach based on Voronoi diagrams.

In a previous study, the authors [17] have proposed an adaptable cluster formation technique that employs Voronoi tessellations to define the cluster zones to be used to track Mobile Nodes in congested noisy environments. The technique partitions the tracking space into a number of regions, each of which are to be monitored by one cluster comprised of a sink node and multiple anchor nodes.

*Ad hoc* deployment of large number of wireless sensor nodes is proposed as an alternative approach to the explicit cluster formation techniques described previously. Due to the unique characteristics and behaviours of *ad hoc* networks, there is a growing interest in developing protocols for *ad hoc* wireless sensor networks including clustering, location discovery protocols, and position based routing protocols among others.

Savarese *et al.* [18] presented a clustering based solution to the problem of location discovery in wireless *ad hoc* networks with no Mobile Nodes or wireless nodes with limited mobility. The proposed localization algorithm is an anchor based method that relies on the *a priori* coordinates of sparsely located anchor nodes. Clusters of nodes surrounding anchor nodes cooperatively establish confident position estimates through assumptions, checks, and iterative refinements. Once established, these positions are propagated to more distant nodes, allowing the entire network to create an accurate map of itself.

Basagni [19] proposes a distributed approach to the clustering of quasi-static and mobile *ad hoc* networks. The proposed approach groups the nodes by following a new weight-based criterion that allows the choice of the nodes that coordinate the clustering process based on node mobility-related parameters. The proposed algorithm is executed at each node with the sole knowledge of the identity of the one hop neighbors.

Table 1 provides a summary of the recent work on wireless sensor node clustering strategies.

**Table 1:** Recent work on wireless sensor node clustering strategies.

Authors	Network Type	Localization	Clustering Technique	Notes
Horling *et al.* [10]	heterogeneous	range-free	pre-defined (static)	Use of software agents for clustering
Bandyopadhyay and Coyle [11]	homogeneous	range-based	pre-defined (static)	Energy efficiency
Yu *et al.* [12]	homogeneous	range-based	pre-defined (dynamic)	Energy efficiency
Sun *et al.* [13]	homogeneous	range-based	pre-defined (static)	Security
Chen *et al.* [15]	heterogeneous	range-free	pre-defined (dynamic)	Voronoi cells
Boukerche *et al.* [16]	heterogeneous	range-based	pre-defined (static)	Voronoi cells
Gholami and Brennan [17]	heterogeneous	range-free	pre-defined (dynamic)	Voronoi cells
Savarese *et al.* [18]	heterogeneous	range-based	*ad hoc*	map-based
Basagni [19]	homogeneous	range-based	*ad hoc*	Weight-based criterion to determine CH

This work has primarily focused on the properties of specific clustering approaches (e.g., energy efficiency, security, *etc.*). Although some work has been done on node clustering strategies that dynamically adapt to changing industrial conditions, very little work has been done on the trade-offs between competing approaches to this problem. In the remainder of this paper, we tackle these open issues by combining a multi-agent approach with the recent work on efficient sensor node partitioning using Voronoi clusters. In the next section, we describe this general approach. Although considerable work has been accomplished on WSN node management, the trade-offs between alternative clustering approaches are not well understood. In this paper, we compare two general approaches that span the different clustering approaches proposed in the literature. In order to assess the adaptability of these approaches, the comparisons are performed in a harsh, industrial environment with a network. In the next section, we provide a description of the general architecture of the WSN used in this research along with an overview of the clustering approaches.

## 3. Distributed Clustering Techniques

### 3.1. The Wireless Sensor Network Architecture

The proposed WSN consists of the three basic types of wireless sensor nodes shown in Figure 1: (1) Sink Nodes; (2) Anchor Nodes; and (3) Mobile Nodes. Sink Nodes are equipped with a powerful processing unit, an RF transceiver unit, a high capacity memory unit, and a long-lasting power supply unit to support two key agent types: (1) a Mediator Agent (MA) that is responsible for cluster formation and management, as well as inter cluster communication; and (2) Locator Agents (LA) that are responsible for Mobile Node tracking. For our proposed approach, a unique LA is assigned to each Mobile Node tracked by the cluster.

Anchor Nodes are responsible for sensing the distance to a particular Mobile Node and transmitting the distance estimations to a Sink Node. It is generally assumed that the location of these nodes with respect to a global frame of reference is either known *a priori* or can be acquired at any point in time. The processing capabilities of these nodes are much less than those of Sink Nodes since only a single agent, the Anchor Node Agent (ANA), is required to interface these nodes with the agent system.

Finally, the third type of wireless sensor node, the Mobile Node, is very similar in capabilities to Anchor Nodes, but by definition, can move from one location to another. Like the Anchor Node, a single interface agent, the Mobile Node Agent (MNA) in this case, resides on the computing platform. Additional details on the design and implementation of the agent architecture are provided in [8] and [20].

### 3.2. Cluster Formation Techniques

In order to provide an objective comparison of alternative distributed cluster formation techniques, we chose to simulate three clustering techniques (Table 2) that approximate the range of techniques from the literature.

**Table 2:** Cluster formation techniques.

Cluster Formation Technique	Protocol	Cluster Head	Notes
Static	Contract Net Protocol (CNP)	Sink Node	Bidding initiate by Sink Node Agent
Dynamic	Vickrey Auction	Sink Node	Bidding initiated by Anchor Node Agent
*Ad Hoc*	Vickrey Auction	Sink Node	Bidding initiate by Sink Node Agent

It should be noted that all three techniques shown in Table 2 are heterogeneous (*i.e.*, are composed of multiple node types) and use range-free localization (*i.e.*, there are no assumptions about point-to-point distances between nodes). As well, all three techniques use bidding-based protocols to recruit nodes to form clusters.

The first two techniques result in pre-defined clusters of Anchor Nodes that are centered on a Sink Node cluster head. For the Static technique, the cluster formation process is initiated by Sink Nodes using the well-established Contract Net Protocol (CNP) for distributed communication and control [21]. In contrast, the Dynamic technique relies on Anchor Nodes to initiate cluster formation using a Vickrey auction [22]. The implementations of the CNP and Vickrey auction protocols are shown in Appendix 1.

The main difference between the static and the dynamic techniques is their ability to adapt to change during operation. Typically, a cluster formation technique is regarded as dynamic when it includes regular (periodic or event-driven) cluster head re-election or cluster reorganization procedures, either to effectively react to network topology changes and adjust appropriately the cluster topology, or simply aiming at the appropriate rotation of the cluster head role among the nodes to gain energy efficiency. Given that our WSN is heterogeneous with Sink Nodes serving as cluster heads, cluster head re-election does not occur. However, the dynamic technique differs from the static technique in its ability to reorganize its clusters to facilitate Mobile Node tracking. As its name implies, clusters formed by the static technique are fixed; in contrast, clusters formed by the dynamic technique can adapt by borrowing Anchor Nodes from adjacent clusters as Mobile Nodes approach cluster boundaries. The intention is to approximate the more dynamic pre-defined clustering approaches of [12,15] with the dynamic technique.

The bidding-based protocols used by both the static and the dynamic techniques result in clusters where each Anchor Node is a member of the cluster whose mean (the Sink Node or “cluster head”) is located closer to the Anchor Node than any other. This process results in a distributed implementation of the k-means algorithm that has the advantage of addressing the computational complexity of the k-means problem [23]: by distributing the algorithm over a network in this manner, our approach allows the clustering problem to be tackled in a reasonable time. Like many of the pre-defined clustering approaches reported in the literature, the computational complexity of the static and dynamic techniques are in the order of *O*(*n*), where *n* represent the number of nodes.

The resulting network topology is a set of clusters in the form of Voronoi cells [24]. For both the static and the dynamic techniques, we use this network topology, along with the average received signal strength (ARSS) to determine when a Mobile Node tracking task is passed from one cluster to the next.

**Figure 2:**
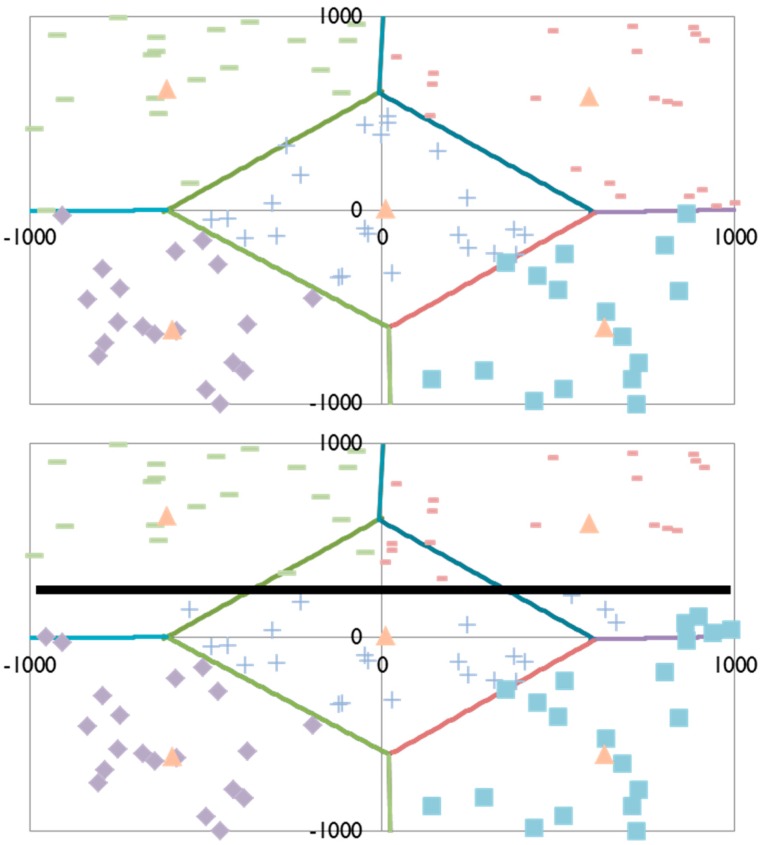
Adaptability of the auction-based cluster formation technique in response to signal blockage.

The bidding-based protocols used for cluster formation also provide the static and dynamic techniques with the ability to adapt to the shop floor environment. For example, the sensor network shown in Figure 2 has been divided into five clusters: each managed by one Sink Node (represented by solid triangles in the figure). In the upper figure, the Anchor Nodes have been assigned to clusters that correspond to Voronoi cells: to identify the clusters, a different shape is used to represent Anchor Nodes within the cluster (*i.e.*, rectangles, crosses, diamonds) and the Voronoi boundaries have been identified by lines. When an obstacle is introduced in the lower figure (the solid horizontal line), the clusters reconfigure in response to the resulting signal blockage. As can be seen in Figure 2, the clusters still conform to a Voronoi pattern wherever possible, but no longer include anchors that are blocked by the obstacle (these anchors are assigned to adjacent clusters).

The third technique shown in Table 2 is intended to approximate the *ad hoc* clustering techniques described in the previous section. For this technique, Sink Nodes again serve the role of cluster heads, but Anchor Nodes are temporary members of clusters that are formed for each Mobile Node tracking task. In order to determine which Sink Node is to serve as cluster head for a given tracking task, Sink Nodes initiate Vickrey auctions amongst themselves. The winning Sink Node then manages the tracking task by recruiting Anchor Nodes into an *ad hoc* cluster. This cluster remains active until either the Sink Node does not have enough resources (e.g., wireless nodes) to track the event (*i.e.*, in case the event is mobile and goes out of the detection range of the *ad hoc* cluster), or the Sink Node decides to no longer track the event. At this point all the participating wireless nodes are dismissed and they will no longer have any commitment to the *ad hoc* cluster (*i.e.*, the transient organization).

## 4. Experimental Section

In this section, we compare the distributed cluster formation techniques described in Section 3 in the context of Mobile Node tracking in an industrial WSN. As noted previously, our aim is to investigate the reconfigurability of alternative clustering approaches, and in particular, how cost effectively each approach adapts to meet new or changing requirements. To perform this comparison, we introduce three performance measures: (1) a cost/efficiency related “load distribution” measure; (2) a performance related “effectiveness” measure; and (3) a resource consumption measure. We follow this with a description of our experimental design in Section 4.2 and the results of our simulation experiments in Section 4.3.

### 4.1. Metrics

A reconfigurable system is generally understood to be a system that can be adapted to meet new or changed requirements. However, as in natural systems (biological, ecological), this adaptability is typically at the cost of efficiency. In order to quantify this trade-off in a distributed WSN, we propose the three basic metrics described in this subsection.

#### 4.1.1. Load Distribution

The first metric, a cost/efficiency related criterion, measures the load distribution among Anchor Nodes. In a computer network, load distribution or load balancing is used to distribute workloads across multiple computing resources, such as computers, central processing units (Sink Nodes), or wireless nodes. Load distribution (balancing) aims to optimize resource use, maximize throughput, minimize response time, and avoid overload of any of the resources.

In this research, we are interested in measuring how efficient the three clustering approaches are at distributing the load among Anchor Nodes. To measure load distribution among Anchor Nodes, we propose utilizing the statistical variance of the number of times each anchor is employed to estimate a distance to a Mobile Node. The variance explains how the workloads are distributed among Anchor Nodes by the cluster. In other words, the lesser the variance the more distributed the workloads among wireless nodes, *i.e.*,

(1) $$f^{lds}=Var\left(X\right)=\;\frac{\sum_{i=1}^{N}{\left(X_{i}-\mu \right)}^{2}}{N-1}\;X_{i}=number\;of\;times\;anchor\;i\;is\;utilized$$

where *N* is the number of Anchor Nodes and μ is the mean of $X_{i}\;i=1,2,\ldots ,N$.

#### 4.1.2. Effectiveness of the Cluster Formation Technique

The second metric is concerned with the performance of the distributed cluster formation techniques, and more specifically, their ability to adapt to the movement of Mobile Nodes through the tracking zone. Since the WSN investigated in this research is composed of stationary sensor nodes (Sink Nodes and Anchor Nodes), this form of adaptability involves “virtual mobility” of these stationary tracking nodes. In other words, as a Mobile Node traverses the tracking zone, the clusters adapt to the tracking task by assigning Anchor Nodes with the closest proximity to the Mobile Node tracking task.

As a metric to measure how effective a given cluster formation is and to compare the performance of the cluster formation techniques in deploying a proper set of Anchor Nodes, we propose using the average distance each Mobile Node is at any point in time from the estimating Anchor Nodes during the course of WSN operation, *i.e.*,

(2) $$\begin{array}{cc} f^{ecf}= & \frac{\sum_{\forall t}\sum_{\forall i}\sum_{\forall j}d_{i,j}^{t}}{TNM}\;i\in \left\{deployed\;mobile\;nodes\right\},j\\ & \;\in \left\{estimating\;anchor\;nodes\right\},\;and\;t\\ & \;\in \left\{quantized\;operation\;time\right\} \end{array}$$

where $d_{i,j}^{t}$ is the distance from Mobile Node *i* to estimating anchor *j* at quantized time *t*, *T* is the WSN/WAN operation period, *N* is the number of Mobile Nodes in the wireless network, and *M* is the number of distance estimating Anchor Nodes.

#### 4.1.3. Resource Consumption

Wireless nodes’ battery and communication bandwidth are two main resources of a WSN every distributed system must sparingly use.

One key restriction on the wireless networks is the limited communication band width, which limits the number of message that can be communicated at a time among a cluster of wireless nodes located at close vicinity with respect to each other. Hence, it, in turn, limits the rate of data transferred among the wireless nodes.

Transmission and receipt of messages also play a significant role in the battery life of a wireless node. In fact, wireless communication—in terms of transmission and receipt of messages—is the major energy consumer during the system operation [4]. This is to say, the higher the rate of communication, the more energy is consumed by the system. Therefore, it is important to consider the communication rate when designing energy-efficient tracking techniques.

Thus, the rate of messages communicated among wireless nodes in a cluster of WSN plays a very crucial role in the practicality of the clustering technique, hence the tracking technique, both in terms of available bandwidth and energy consumption of wireless nodes.

(3) $$f^{tmg}=\;\frac{\sum^{\text{​}}messages}{period}\;\left[msg\cdot s^{-1}\right]$$

#### 4.1.4. Tracking Accuracy

Given the actual location of a Mobile Node, *i*, at a given time, the accuracy of the location estimation, *f^acc^*, is measured by the Euclidean distance between the estimated location of the Mobile Node (*x^e^*, *y^e^*) and its actual location (*x^a^*, *y^a^*) at any point in time during the course of simulation, *i.e.*,

(4) $$f_{i,t}^{acc}=\sqrt{{\left(x_{i,t}^{a}-x_{i,t}^{e}\right)}^{2}+{\left(y_{i,t}^{a}-y_{i,t}^{e}\right)}^{2}}$$

### 4.2. Experimental Design

A simulator is used to examine the proposed cluster formation techniques in dynamic and obstructed environments typical of industrial environments. Selecting a set of parameters of interest for further studies, statistical design of experiments is also used to analyze the sensitivity of these parameters to changes and fluctuations.

In this paper, we take into consideration the following ambient and medium related factors as well as a selection of design factors for further investigations into their impact on the WSN clustering techniques: number of Sink Nodes, number of Anchor Nodes, signal blockage level, and cluster formation techniques. Table 3 summarizes these factors and the levels that they can take. The choice and level of the factors used for these experiments were determined through experimental work with a 433 MHz MICA2 based Cricket platform, which is comprised of six Cricket motes (at least one of which was mobile). This work is reported in [25].

**Table 3:** Experimental factors and their levels.

Factor	Levels	Description
No. Sink Nodes (A)	A1	4
	A2	7
No. Anchor Nodes (B)	B1	400
	B2	676
Signal blockage % (D)	D1	0%
	D2	5%
Cluster formation technique (E)	E1	*Ad hoc*
	E2	Dynamic
	E3	Static

To study the adaptability of the cluster formation techniques, we limit the effect of ambient conditions to different levels of signal blockage. In a previous study, we tested the effect of other ambient conditions such as varying temperature on the wireless sensor networks, and concluded that air humidity, pressure and temperature have measurable but negligible impact on the wireless sensor networks [8].

Given the factors introduced in Table 3, we can devise a statistical scheme for the experiments as follows. We have four factors in total, three of which have two levels and one has three levels. With a full factorial design and no blocking (one block), we will have the following design [26]:

$$2^{3}\times 3^{1}$$

This design has 24 base runs; with only four replications for every treatment and two additional replications for every run of E2 and E3 levels, the total number of experiments is 160.

To reduce the effects of external factors—either in the form of noise or disturbance—randomization of the running order of the treatments are widely suggested [26]. In this study, we randomize running order of the resultant treatments via Minitab 16 software. The design table is constructed in Minitab to produce a standard run order; however, to reduce the potential influence of any external factors, the run order is randomized to result in a randomized run order to be used to execute the experiments accordingly.

### 4.3. Experimental Results

A JADE (Java Agent DEvelopment framework [27]) based simulator was employed to run 160 experiments based on the experimental design introduced in the Section 4.2; then, the results obtained were evaluated against the criteria introduced in Section 4.1. In the sections that follow, we report and analyze the results obtained.

Normal probability and histogram plots were used to examine the normal distribution of the obtained response data. For all sets of experiments under each of two criteria, to an acceptable level of approximation, the obtained response data follow a normal distribution. Moreover, for all sets of experiments under each of two criteria, the residual plots do not illustrate any patterns of anomaly.

**Figure 3:**
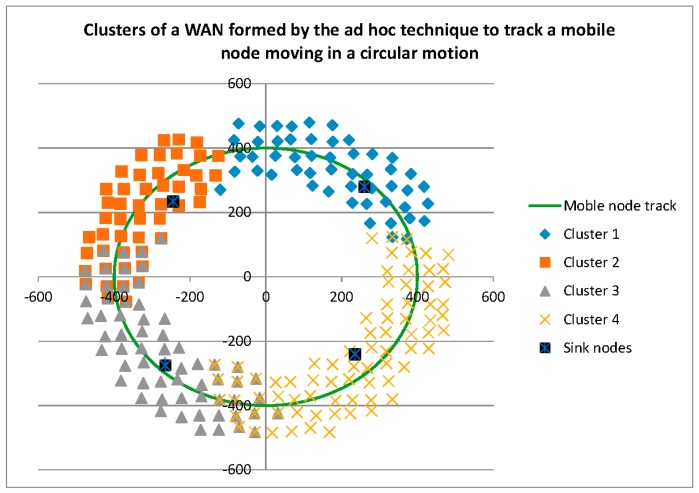
Clusters formed by the *Ad Hoc* technique.

**Figure 4:**
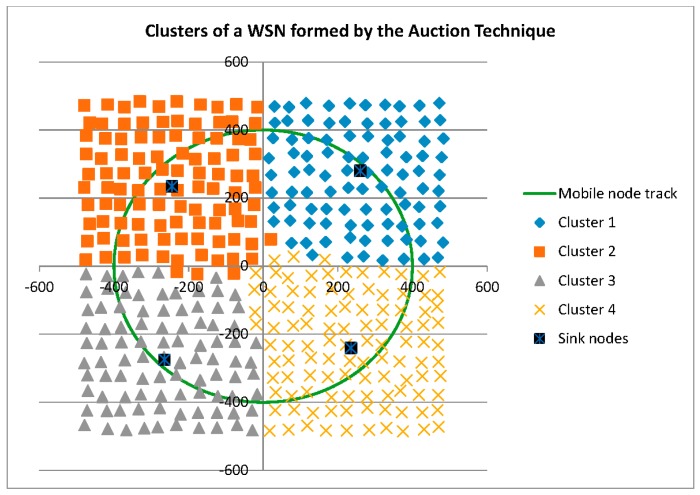
Clusters formed by the Dynamic technique.

**Figure 5:**
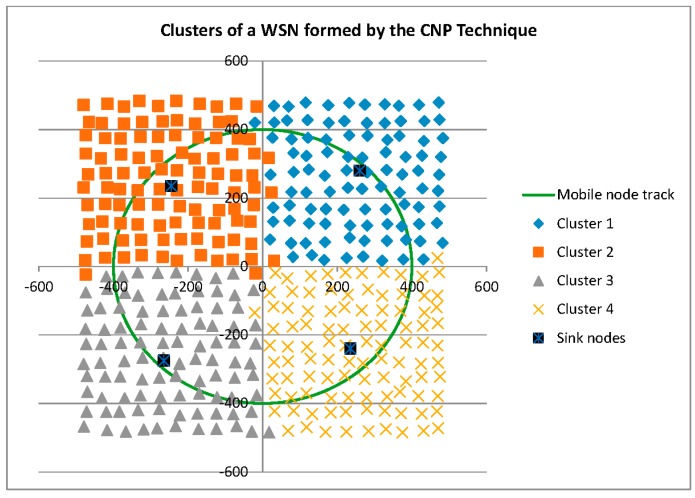
Clusters formed by the Static technique.

Before reporting on the reconfigurability and performance related measures, it is interesting to graphically compare the three cluster formation techniques. As shown in Figure 3, the *ad hoc* technique recruits only those Anchor Nodes required for tracking Mobile Nodes, and shares Anchor Nodes between cluster heads (Sink Nodes) as can be seen by the overlapping Anchor Nodes in this figure. The dynamic and static techniques, shown in Figure 4 and Figure 5, respectively, result in the same Voronoi cell pre-defined clusters.

In the remainder of this section, we summarize the results for the four experimental factors in Table 3. A summary of the statistical results of our experiments is provided in Table 4.

**Table 4:** Statistical results for the WSN simulations.

Metric	Significant at *p < 0.05*			
	Number of Sink Nodes	Number of Anchor Nodes	Blockage	Cluster Formation Technique
Load Distribution	No	Yes	Yes	Yes
Effectiveness	Yes	Yes	Yes	Yes
Resource Consumption	Yes	Yes	Yes	Yes
Tracking Accuracy	Yes	No	No	Yes

#### 4.3.1. Load Distribution

**Figure 6:**
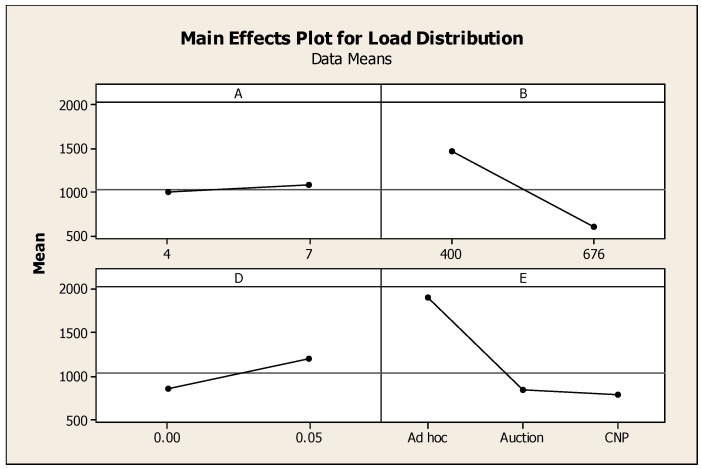
Main effect plot for load distribution. *n* = 160, ANOVA, 1 *df*, *p* < 0.05.

As noted in Section 4.1.1, the load distribution measure explains how workloads are distributed among Anchor Nodes: the lesser the metric (variance), the more distributed the workload is among Anchor Nodes. For this metric, both the number of Anchor Nodes and the level of blockage are significant: *i.e.*, as would be expected, larger numbers of Anchor Nodes and lower levels of signal blockage allow the workload to be distributed more evenly among nodes. The results for the main effects of the load distribution parameter are summarized in Figure 6.

The main effect plot shown in Figure 6 is used to examine the differences between the level means for the factors listed in Table 3. For this plots, a horizontal line indicates that each level of the factor affects the response in the same and, as a result, there is no main effect. For example, the near horizontal line shown in the upper left corner of Figure 6 for Factor A, “number of sink nodes”, show load distribution is not affected by a change in the number of sink nodes. However, when the line is not horizontal, there is a main effect and the steeper the slope of the line, the greater the magnitude of the main effect. For example, the large slope shown in the upper right corner of Figure 6 for Factor B, “number of anchor nodes”, shows that load distribution is affected by the number of anchor nodes.

**Figure 7:**
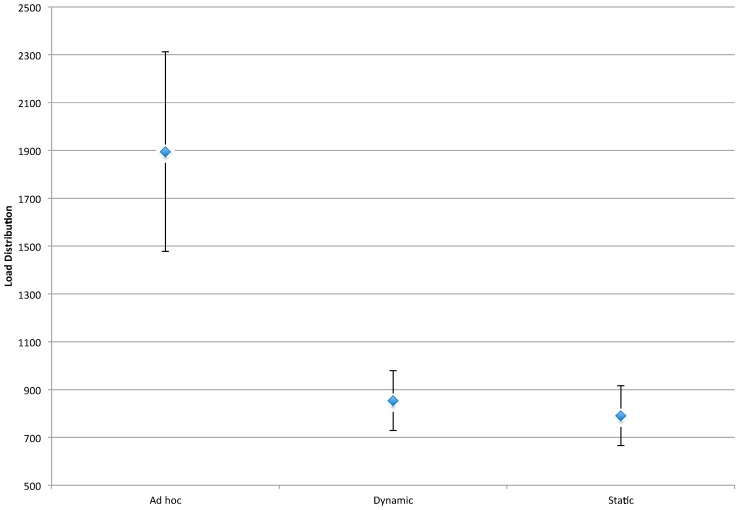
Interval plot for load distribution. *n* = 160, ANOVA, 1 *df*, *p* < 0.05.

As shown in Figure 7, the pre-defined clusters outperform the *ad hoc* technique for this metric. A brief inspection of Figure 3, Figure 4 and Figure 5 may lead one to see this as an obvious result: *i.e.*, the larger number of Anchor Nodes shown in the pre-defined figures (Figure 4 and Figure 5) should result in better Anchor Node load distribution. However, Figure 4 and Figure 5 show the Anchor Nodes recruited into each pre-defined cluster: they do not show the Anchor Nodes actually used for the circular tracking task. The results of Figure 7 show that Sink Nodes in the Static and Dynamic techniques actually spread the workload among Anchor Nodes more evenly than Sink Nodes in the *ad hoc* technique. This is a result of the inter-cluster trade-off protocols used by the pre-defined clustering techniques: *i.e.*, a tracking task is not transferred to an adjacent cluster until the Mobile Node reaches the boundary of the cluster; alternatively, tracking tasks are transferred much earlier with the *ad hoc* technique as can be seen by the broader “boundaries” between clusters (*i.e.*, shared Anchor Nodes) in Figure 7.

**Figure 8:**
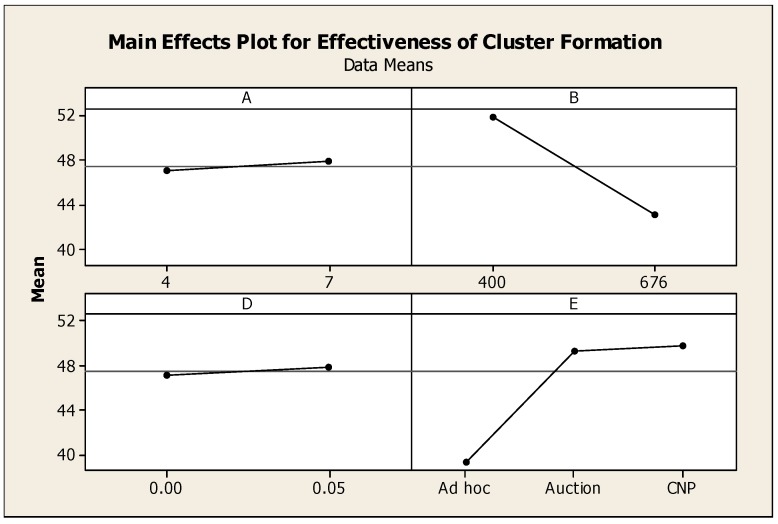
Main effect plot for effectiveness. *n* = 160, ANOVA, 1 *df*, *p* < 0.05.

#### 4.3.2. Effectiveness of the Cluster Formation Techniques

For the purposes of this study, effectiveness is a measure of the average distance between a Mobile Node and the Anchor Nodes that track it: the lower the average distance, the more effective the cluster formation technique. For this metric, both the number of Anchor Nodes and the cluster formation technique are significant. More specifically, the greater the number of Anchor Nodes, the shorter the distance between Mobile Nodes and tracking Anchor Nodes (due to a higher concentration of Anchor Nodes in the network). This result is shown in the main effect plot for effectiveness in Figure 8.

**Figure 9:**
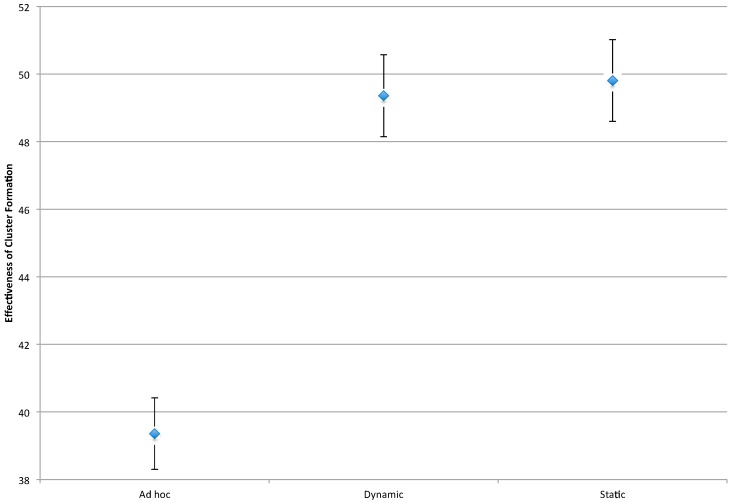
Interval plot for effectiveness. *n* = 160, ANOVA, 1 *df*, *p* < 0.05.

As shown in Figure 9, of the three clustering techniques, the *ad hoc* technique results in the closest spacing between Mobile Nodes and their tracking Anchor Nodes. This result is supported by Figure 3, which shows a tight clustering of Anchor Nodes around the Mobile Node path.

#### 4.3.3. Resource Consumption

**Figure 10:**
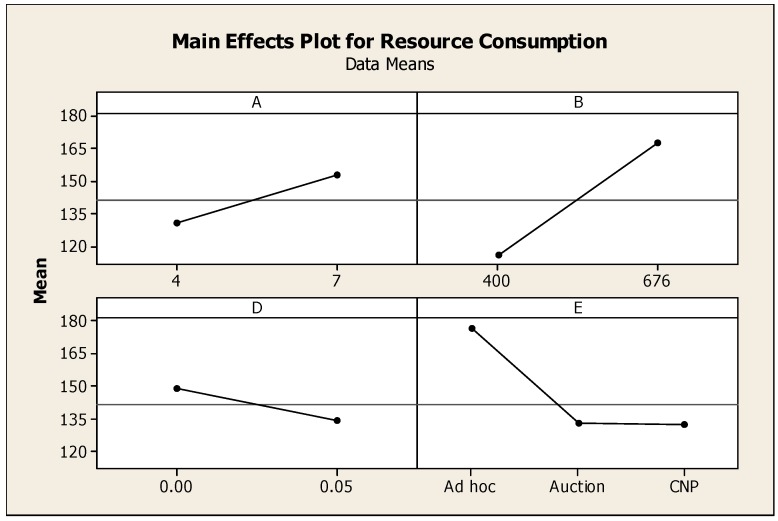
Main effect plot for resource consumption. *n* = 160, ANOVA, 1 *df*, *p* < 0.05.

As noted in Section 4.1.3, resource consumption is represented by the rate of messages exchanged between sensor nodes: *i.e.*, as message rate increases, processor load and battery consumption increases. For this measure, all of the experimental factors described in Section 4 are significant. The main effect plot for this measure is shown in Figure 10.

The results for the first two factors are a direct consequence of increasing the number of participants in the bidding process. More specifically, as the number of nodes (Sink Nodes and Anchor Nodes) increase, more CNP and Vickrey auction messages are sent. The third factor, signal blockage, impacts the number of messages received: *i.e.*, as blockage increases, signal paths are limited, and consequently, message exchange decreases.

**Figure 11:**
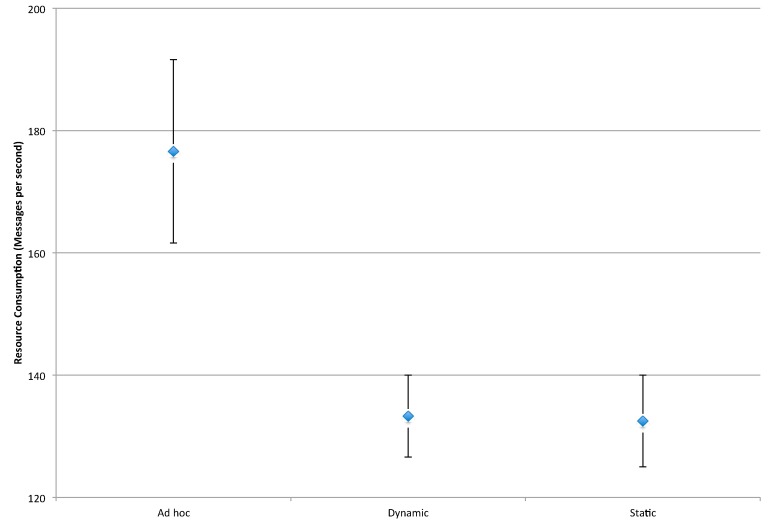
Interval plot for resource consumption. *n* = 160, ANOVA, 1 *df*, *p* < 0.05.

Figure 11 provides a comparison of the clustering techniques for this measure. As can be seen in this figure, the *ad hoc* approach results in the highest message rate, and as a result, the greatest consumption of network resources. This heavy resource consumption is exacerbated by the load distribution and effectiveness results described previously. More specifically, in addition to resulting in a higher number of messages per second to support its protocol, the *ad hoc* technique involves fewer Anchor Nodes in these communications. Given the direct link between communications and battery life, the *ad hoc* technique will result in a much shorter mean time between failure of sensor nodes, than the two pre-defined clustering techniques.

#### 4.3.4. Tracking Accuracy

**Figure 12:**
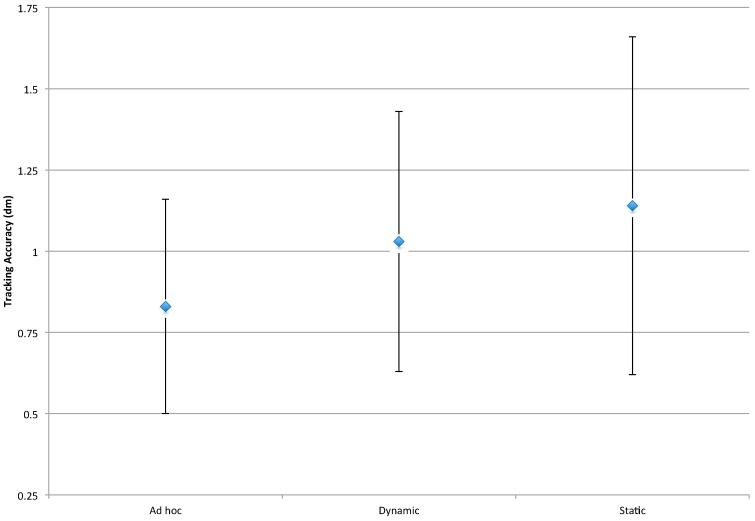
Interval plot for tracking accuracy. *n* = 160, ANOVA, 1 *df*, *p* < 0.05.

Figure 12 provides a comparison of the three techniques on the basis of tracking accuracy. Given that our measure of tracking accuracy is based on the Euclidean distance between estimated and actual location, the lower the measure, the better the tracking accuracy. As can be seen in this figure, tracking accuracy improves with the relative adaptability of the clustering technique. Comparing the Static technique with its fixed clusters to the dynamic technique with its ability to share Anchor Nodes between clusters, we see an improvement in tracking accuracy. Similarly, the *ad hoc* technique’s ability to freely form clusters results in the best tracking accuracy.

These results are a consequence of the quality of service issues that occur as Mobile Nodes reach the boundaries of sensor node clusters. For example, fixed clustering techniques like the static technique can result in deteriorating conditions (lost estimations, weak signals, *etc.*) as Mobile Nodes reach the boundary of the tracking cluster since they only deploy Anchor Nodes within their cluster; in contrast, a very adaptable technique like the *ad hoc* technique does not have any restrictions concerning Anchor Node commitments to clusters when choosing among available Anchor Nodes.

## 5. Conclusions and Future Work

In this paper, we compare distributed clustering techniques for wireless sensor networks on the basis of their reconfigurability, and in particular, on the basis of their ability to adapt to changes and disturbances during Mobile Node tracking. The clustering techniques are intended to span a range of pre-defined clusters based on efficient geographic partitioning of sensor nodes around cluster heads (in this case, in the form of Voronoi cells) and *ad hoc* clusters that are formed “on the fly” during the tracking task.

All three of the clustering techniques showed that they are capable of reconfiguring their structures to adapt to changing shop floor and Mobile Node tracking conditions. However, as would be expected given the basic designs of the three techniques, the *ad hoc* approach resulted in better cluster formation effectiveness; in other words, the *ad hoc* approach appears to be more adaptable to the Mobile Node tracking task than the pre-defined clustering approaches. This higher effectiveness of the *ad hoc* approach is at the cost of poorer load distribution though. As a result, the trade-off for higher adaptability in this case is the potential for an unbalanced depletion of resources (*i.e.*, wireless node battery life) and, correspondingly, unpredictable performance of the overall network.

In this paper, we merely focused on distributed dynamic cluster formation techniques as a solution to the reconfigurability and adaptability required of a tracking system to position/localize mobile wireless sensor nodes in factory environments. However, distributed dynamic clusters are only one component of such a tracking system. Details of network architectures and protocols as well as the multi-agent system to manage such a tracking system are to be reported in our future papers.

Our current work in this area is focused on developing a multi-agent cluster management system to encourage maintenance by reclaiming and replacing nodes over time. For this work, we are investigating the use of homogeneous wireless sensor networks that provide minimal setup and changeover effort due to their hardware indifferences.

## Figures and Tables

**Figure A1:**
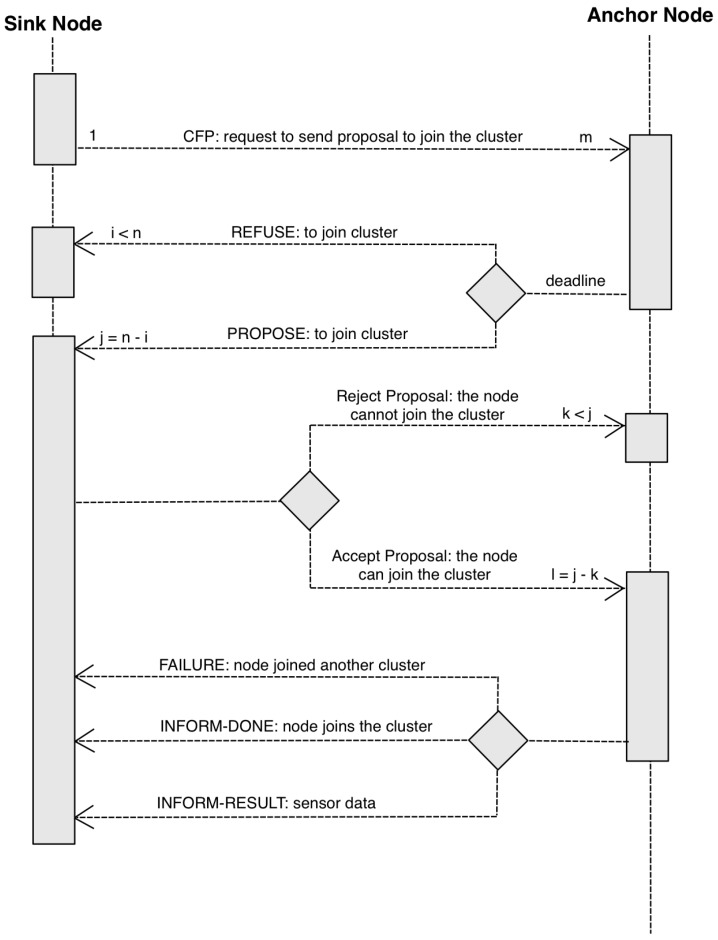
Agent interaction diagram showing distributed cluster formation using the static technique.

**Figure A2:**
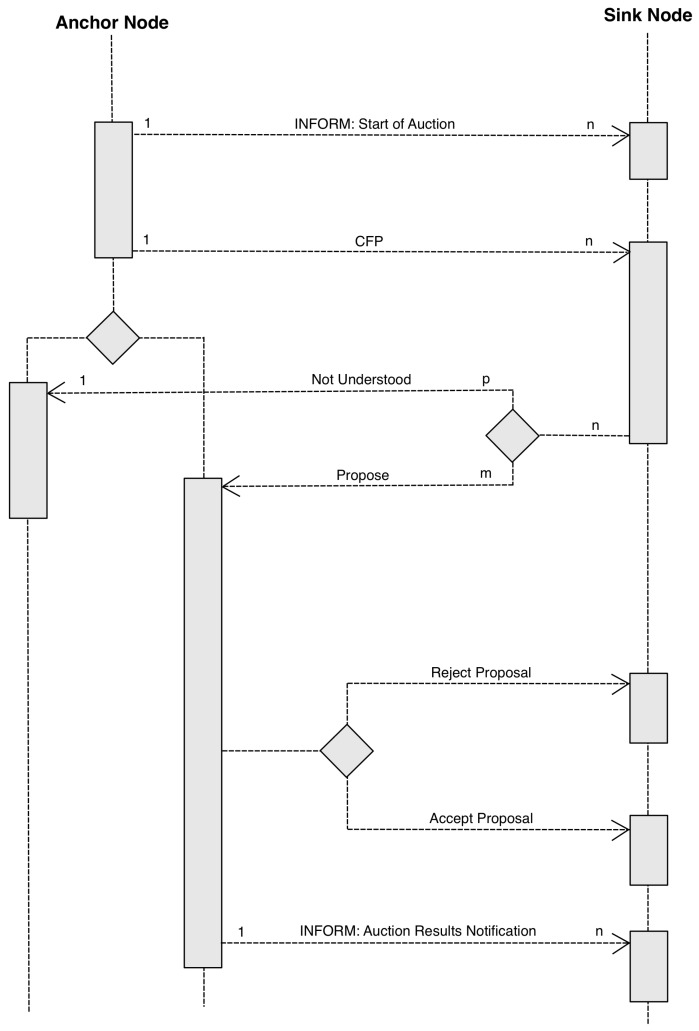
Agent interaction diagram showing distributed cluster formation using the dynamic technique.
